# Management of post-traumatic stress disorder symptoms by yoga: an overview

**DOI:** 10.1186/s12906-023-04074-w

**Published:** 2023-07-21

**Authors:** Nina Laplaud, Anaïck Perrochon, Matthieu Gallou-Guyot, Maarten Moens, Lisa Goudman, Romain David, Philippe Rigoard, Maxime Billot

**Affiliations:** 1 ILFOMER (Institut Limousin de FOrmation Aux Métiers de La Réadaptation), Université de Limoges, Limoges, France; 2 Laboratoire HAVAE, Université de Limoges, 20217 Limoges, UR France; 3 Department of Neurosurgery, Universitair Ziekenhuis Brussel, Laarbeeklaan 101, 1090 Brussels, Belgium; 4 STIMULUS Research Consortium (Research and TeachIng Neuromodulation Uz Brussel), Vrije Universiteit Brussel, Laarbeeklaan 103, 1090 Brussels, Belgium; 5 Center for Neurosciences (C4N), Vrije Universiteit Brussel, Laarbeeklaan 101, 1090 Brussels, Belgium; 6 Department of Physiotherapy, Pain in Motion (PAIN) Research Group, Human Physiology and Anatomy, Faculty of Physical Education and Physiotherapy, Vrije Universiteit Brussel, Laarbeeklaan 103, 1090 Brussels, Belgium; 7 Department of Radiology, Universitair Ziekenhuis Brussel, Laarbeeklaan 101, 1090 Brussels, Belgium; 8 Research Foundation—Flanders (FWO), 1090 Brussels, Belgium; 9 Department of Physical and Rehabilitation Medicine, Poitiers University Hospital, University of Poitiers, 86000 Poitiers, France; 10 PRISMATICS (Predictive Research In Spine/Neurostimulation Management And Thoracic Innovation in Cardiac Surgery), University Hospital of Poitiers, Poitiers, France; 11 Department of Spine Neurosurgery & Neuromodulation, Poitiers University Hospital, 86000 Poitiers, France; 12 ISAE-ENSMA, Pprime Institute UPR 3346, CNRS, University of Poitiers, 86000 Poitiers, France

**Keywords:** Complementary and alternative approach, PTSD, Mind–body therapies, Mindfulness, Meditation, Physical activity

## Abstract

**Background:**

Posttraumatic stress disorder (PTSD) can occur after trauma. While PTSD management strategies include first-line pharmacotherapy and psychotherapy, mind–body therapies, such as yoga, are applied in the PTSD population. This overview aimed to summarize the effectiveness of yoga interventions on PTSD symptoms in adults in a systematic review (SR) including randomized controlled trials (RCTs).

**Method:**

We searched for SR with or without meta-analysis of RCTs involving adults with PTSD diagnosis or trauma history. The search was conducted until April 2022, through six databases (Cochrane Database, MEDLINE (Pubmed), Scopus, Embase, CINHAL and PEDro). The primary outcome was the evolution of PTSD symptoms throughout the intervention. Secondary outcomes included follow-up, safety, adherence, and cost of the intervention. Two authors independently performed the selection, data extraction and risk of bias assessment with the AMSTAR 2 tool and overlap calculation. This overview is a qualitative summary of the results obtained in the selected studies.

**Results:**

Eleven SRs were analyzed, of which 8 included meta-analyses. The overlap between studies was considered very high (corrected covered area of 21%). Fifty-nine RCTs involving 4434 participants were included. Yoga had a significant small-to-moderate effect-size on PTSD symptom decrease in 7 SRs and non-significant effects in 1 SR with meta-analysis. All SR without meta-analysis found beneficial effects of yoga on PTSD. Secondary outcomes were not sufficiently assessed to provide clear evidence. Results should be interpreted with caution as 1 SR was rated as at moderate risk of bias, 3 as low and 7 as critically low.

**Conclusions:**

While yoga therapy seems promising for decreasing PTSD symptoms, future research should standardize yoga therapy duration/frequency/type and consider long-term efficacy to better delineate yoga therapy efficacy in PTSD patients.

**Supplementary information:**

The online version contains supplementary material available at 10.1186/s12906-023-04074-w.

## Introduction

Post-traumatic Stress Disorder (PTSD), is diagnosed when intrusion, avoidance, alterations in cognition and mood, alterations in arousal and reactivity last for more than a month [1, 2], can occur after a trauma, such as exposure to death or to a death threat, to serious injuries or to sexual violence [1]. Considering all origins of the trauma, 4% of the population risk developing PTSD worldwide [2] leading to psychological distress, social impairments and alteration of global health [1, 3]. In attempts to improve the challenging management of PTSD symptoms, which is conventionally treated by psychotherapy and pharmacotherapy [4–7], complementary approaches including yoga are nowadays well-considered [8].

Initially practiced to cultivate an inner state of equanimity, and to reach a higher level of consciousness [9, 10], yoga is currently practiced mainly as a way to promote physical activity and mental well-being [10, 11]. Yoga is based on the practice of three main principles with physical postures, breathing techniques and meditation [10–13] and is carried out by more than 300 million people around the world. Yoga claims to provide health benefits including physical, metabolic, physiological, mental health and well-being in the general population [14, 15] and in populations presenting with psychological impairments [13]. In an overview including 13 systematic reviews (SR) and 1 meta-analysis published in 2015, Macy and al. [16] synthesized the impact of yoga on mental health problems. While the authors reported overall positive results on PTSD symptoms, the level of evidence was limited by the inclusion of clinical trials both with and without a controlled randomized design. The new recommendations of the International Society for Traumatic Stress Studies emphasized a need for additional studies to determine the efficacy of yoga on PTSD symptoms [7]. In a recent bibliometric analysis of SR, Wieland et al. [13] highlighted the growing interest in yoga therapy and retrieved 332 SR of which 8 specifically focused on PTSD. The authors recommended examining the quality of SRs regarding yoga, the objective being to establish a new global synthesis of literature by providing current evidence of yoga efficacy on PTSD symptoms and recommendations for future research.

To achieve this goal, we conducted an overview of SR including RCTs only. The main aim was to provide current evidence of the effect of yoga treatment on PTSD symptoms. In addition, retention of benefits, safety, adherence and cost were considered as secondary outcomes likely to provide recommendations for future studies.

## Method

### Design and protocol

Our overview was conducted following the Cochrane recommendations [17] and the Preferred Reporting Items for Overviews of SRs Including Harms (PRIO-harms) [18]. The PRIO-harms tool is a modified version of PRISMA recommendations concerning SRs [19]. PRIO-harms recommendations aim to structure an overview through 27 items.

### Search strategy

To ensure screening a maximum amount of items in the literature, and to avoid missing out on any SR, six databases were consulted the Cochrane Database, MEDLINE (Pubmed), Scopus, Embase, CINHAL and PEDro. The search was performed up until April 2022. Keywords in titles and abstracts were used to select the SRs: yoga AND posttraumatic stress disorder (see details in Appendix 1). Keywords were selected with MeSH terms to ensure the inclusion of synonyms in our research. In addition, grey literature was considered in the present overview.

### Eligibility criteria and selection

The research question and eligibility criteria were defined following the PICOS (*Population/Patient/Problem*, *Intervention*, *Comparison*, *Outcome*, *Study design*) method [20]. The inclusion criterion were SRs assessing the effectiveness of yoga intervention on PTSD symptoms of affected adults, as the primary outcome, in comparison with active control groups or no intervention. Changes in PTSD symptoms had to be reported with scales or quantified data. SR**s** were included only when the results of yoga were reported with independent analysis, even though other interventions were reported. No restriction on trauma type or on type of yoga intervention was considered. SRs had to include Randomized Controlled Trials (RCTs) only and had to be entirely in English. Study selection was performed by two independent reviewers (NL and MB) in four steps: removal of duplicates, reading of the titles, reading of the abstracts and integral reading. Disagreements between reviewers were solved by discussion and if necessary, with a third independent reviewer (AP). Microsoft Excel ® and Zotero ® software were used to save every step of research.

### Data extraction

Data extraction was conducted independently by two authors (NL and AP), and disagreements were resolved with a third independent author (MGG).

The following information was collected:

- Metadata: first author’s name, publication date, country, type of study, number of primary studies included
- Objectives of the study
- General characteristics of the population: number of participants, age, gender, type of trauma
- Intervention details: type of intervention, control group, duration, frequency of intervention, scales used, first outcome measurement, additional outcomes
- Results after intervention
- Conclusions of first and secondary outcomes

If complementary information was needed, the authors of the selected studies were contacted.

### Study quality assessment

All studies included were evaluated with the AMSTAR 2 scale (A MeaSurement Tool to Assess systematic Reviews) [21]. Two reviewers (NL and MGG) independently read and assessed the risk of bias for each study. Discrepancies were resolved by discussion or with a third reviewer if needed (AP) and noted to report the disagreement rate.

### Overlap

When similar primary studies were included, called overlap, we calculated the corrected covered area (CCA), to avoid any risk of attributing a disproportionate power to the conclusions of a given study [22].

## Results

### Search results

**Fig. 1:**
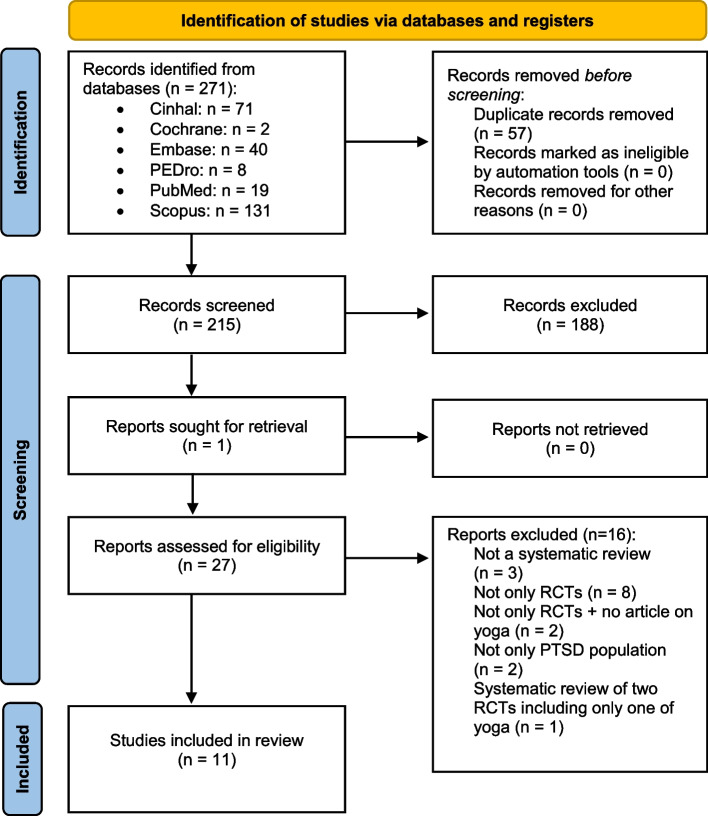
Flow chart

A total of 271 records were identified. After removing 57 duplicates and screening eligible criterion in 27 SRs, 11 articles were included and analyzed. Figure 1 presents the flow chart of the selected reviews, and justifications for excluded reviews are detailed in Appendix 2.

### Description of the included studies

SRs were published between 2015 and 2022, including 3 to 22 RCTs dated from 1985 to 2020. Eight SRs were supplemented by a meta-analysis [3, 23–29]. One SR was presented as a short communication [23]. The CCA was 21%, showing a very high overlap.

### Participants and interventions

The characteristics of the 4434 participants included in the 11 SRs are summarized in Table 1.

**Table 1:** Characteristics of the included reviews

**Review**	**Study design**	**N of primary studies** **(N of yoga studies)**	**N Sexe ratio (% women) Age**	**Population (N RCTs)**	**Yoga interventions (N RCTs)**		**Duration** **Frequency** **N of sessions** **Time of session**	**PTSD** **outcomes (N)** **Others (N)**	**Control group**	**Specific results of yoga**
					**Other interventions (N RCTs)**					**Mains results and conclusions**
**Bisson et al** **2020 UK**[23]	SR + MA	30 (5)	1828 [15–191] n.r n.r	Veterans (7) Military (7) Civilians with PTSD (14) Children ^a^ (2)	TiY (1) KrY (2) SKY (2)		n.r	n.r	WLC/TAU (4) TAU (1)	Low QOE for yoga benefits on PTSD symptoms
					MBSR (5) MR (3) TMS (3) Acupuncture (2) Neurofeedback (2) Saikokeishikankyoto (1) Somatic experiencing (1) Other (8)		n.r	n.r	WLC/TAU (10) WLC (4) PCT (4) Sham TMS (3) WLC/CBT-TF (2) Paroxetine (1) Psychoeducation (1)	Emerging evidence for 6 non- pharmacological and non-psychological interventions for the treatment of PTSD in adults (yoga, acupuncture, neurofeedback, somatic experiencing, saikokeishikankyoto and TMS) All interventions excepted yoga have low QOE
**Björkman et al** **2021** **Sweden **[24]	SR + MA	10 (5)	605 [38–338] 57% Mean age^b^: 45.5 yo	Veterans (2) Veterans and active-duty personnel (1) Veterans and civilians’ women (1) Civilians with PTSD (4)	Online hatha yoga (1) KY (1) TSY with KrY (1) KrY (1) TIY (1)		8—12 weeks Weekly – 2/week 10—20 sessions 1—1.5 h	PCL (2) CAPS (2) ies (1) Depression, anxiety, sleep behavior, dissociative symptoms	WLC / TAU (1) WLC (1) Attention placebo + TAU (1) Attention placebo (1) Assessment only control (1)	No significant differences between low- or high-intensity activities (yoga versus other exercises)
					Aerobic and resistance training (1) Aerobic and anaerobic (2) Resistance training (2)		3—20 weeks Weekly—3/week 9—26 sessions 30 min—1 h	PCL (2) CAPS (1) HTQ (1) PDS (1) Depression, anxiety, QOL, sleep behaviour, substance abuse	TAU (2) WLC (1) WLC / TAU (1) Attention placebo (1)	Positive significant effect of exercise on PTSD symptom severity compared to non-active treatment; high heterogeneity in the results Significant positive effects of exercise for depression, sleep, reduced substance abuse, and increased QOL. No significant result for anxiety Exercise can be included as a part of PTSD treatment
**Cramer et al. 2018** **Germany** [25]	SR + MA	7 (7)	385 [21–100] 46.2% [28.7–58] yo	Veterans (3) Active military and veterans (1) Civilians with PTSD (3)	SKY (2) KY (1) KRY (2) SatY (1) TIY (1)		7 days – 6 months Monthly – daily 7—32 sessions 1 h – 4.4 h	PCL (7) CAPS (3) TLEQ (1) IES (1) DES (1) DTS (1) Retention Safety	WLC (4) Attention control intervention (2) No treatment (1)	Low QOE in favor of yoga compared to no treatment Very low QOE for no differences of yoga compared to attention control interventions Very low QOE for no difference of retention between yoga and both types of control 1 adverse event
**Gallegos et al. 2017 United states** [26]	SR + MA	19 (4)	1173 n.r n.r	Veterans (14) Civilians with PTSD (5)	KrY (1) TIY (1) KY (1) MBX (1)		6 – 10 weeks Weekly—2/week 8—16 sessions 1 h – 1.5 h	CAPS (7) PCL (6) PVSDS (1) IES (1)	WLC (2) SWHE (1) ACG (1)	Marginally significant effects of yoga due to low statistical power and heterogeneity in treatments effects
					Mindfulness (9): MBSR (8), MBB (1) Other meditations^c^ (6) including SKY (2) ^d^ Combination of mindfulness and meditative practices (1)		5 days – 12 weeks 2/week – Daily 6—22 sessions 20 min—6 h	PCL (3) CAPS (1)	TAU (3) WLC (3) PCGT (3) Individual psychotherapy (1) Prolonged Exposure (1) PTSD education group (1) Sitting quietly (1) Sleep hygiene (1) Telehealth psychoeducation (1)	Overall small to moderate ES in favor of meditation and yoga interventions No differences between intervention types, study population, outcome measures, or control condition Meditation and yoga are promising complementary approaches and can be provided as second-line treatment in PTSD
**Hilton et al. 2017** **USA** [27]	SR + MA	10 (3)	643 [28–146] 32.9% [41,–59] yo	Military (6) Civilians with PTSD (4)	KY (1) KrY (1) TIHY (1)		8 – 12 weeks Monthly – weekly 8—14 sessions 1 h – 1.5 h	PCL (2) CAPS (1) Anxiety Depression Adverse events	WLC + TAU (2) TAU, waitlist, women’s health education (1)	Yoga has significant effects on PTSD symptoms and on depression Metaregression: no systematic differences among intervention types on PTSD outcomes (MR/yoga/MBSR)
					MBSR (5) MR (2)		4 – 8 weeks Weekly – 2/week 6—9 sessions 26 min – 2.5 h	CAPS (5) PCL (7) Anxiety Depression QOL Adverse events	TAU (3) WLC + TAU (2) PCGT (1) Psycho-education telehealth (1)	Low QOE for significant decrease in PTSD symptoms of all adjunctive interventions compared to any control No adverse events and no significant results for anxiety
**Kysar-Moon et al** **2021** **USA** [28]	SR + MA	3 (3)	152 [38–64] 100% [18–70] yo, mean age 41.7 yo	Veterans and civilian women (1) Civilians with PTSD (2)	TSY (1) TIY (1) Trauma-focused yoga (1)		6—12 weeks Weekly – 2/week 10—12 sessions n.r	CAPS (1) PCL (1) n.r (1) Depression	WLC (1) SWHE (1) ACG (1)	No significant effects on PTSD symptoms No significant effects on depression symptoms
**Rosenbaum et al** **2015** **Australia** [3]	SR + MA	4 (2)	200 [17–81] n.r [34–52] yo	Civilian with PTSD (3) Veteran (1)	KrY (1) TIY (1)		6—12 weeks Weekly – 2/week 10—12 sessions 1 h—1.15 h	PSS-I (1) CAPS (1) Depression	Health education (1) No treatment (1)	No specific result for yoga intervention
					Combined aerobic and resistance-based intervention (1) Aerobic intervention (stationary cycling) (1)		n.r n.r 12 sessions n.r	PSS-I (1) PCL (1) Depression Cardiovascular risk	TAU (2)	Physical activity significantly reduced PTSD and depressive symptoms compared to control Insufficient datas for anthropometric measures
**Zhu et al.,** **2022** **China** [29]	SR + MA	16 (9)	871 [21–116] 45.5% [18–65] y.o	Adults with PTSD	SKY (2) KrY (2) KY (1) SatY (1) TiY (1) Hatha yoga (1) Online yoga (1)		1—16 weeks Weekly—daily 5 – 32 sessions 1 h—4.4 h	PCL (7) CAPS (3) IES (1) Anxiety Depression	Regular daily life (4) No treatment (2) TAU (1) Mandatory ordinary assistance protocol (1) Toning exercise (1)	No specific result for yoga intervention
					MBSR (4) MBX (1) Brief mindfulness training (1) Integrative exercise (1)		4—8 weeks Weekly—daily 4—64 sessions 1—2.5 h	PCL (5) CAPS (1) BRUMS (1) Anxiety Depression	TAU (3) Regular daily life (2) WLC (1) PCGT (1)	Mind–body exercises had significant effects on PTSD symptoms, depression, and anxiety in patients with PTSD
**Liu et al** **2018** **USA** [30]	SR	13 (3)	953 [29–226] n.r (yoga = 100%) n.r	Veterans (5)Veteran civilian adult women (2)Civilians including (6) adults, nurse, refugees, women with chronic PTSD, children^a^	KrY (2) TIY (1)		10 weeks^b^ Weekly^b^ 10—20 sessions 1 h -1.15 h	CAPS (2) PCL (1) DTS (1) DES (1)	ACG (2) Women’s health education classes (1)	2/3 RCTs found significant difference after yoga intervention None of the studies provided power analysis calculations for primary outcomes
					Mindfulness/ meditation (7) Spiritually based intervention (1) Acupuncture (1) Relaxation training (1)		8 weeks – 12 weeks Weekly—2/week 1 to 16 sessions 20 min to 7 h	PCL (7) CAPS (4) PTSD SSS (1) PSS-SR (1) Researcher-devised self-report assessment of PTSD (1) Biological levels (1)	TAU (2) DI (1) WLC (1) No treatment (2) Psychoeducation (1) PCGT (1) CBT + WLC (1) Exposure + EMDR (1)	Integrative body-mind-spirits interventions have positive effects for treating PTSD
**Niles et al** **2018** **USA** [31]	SR	22 (6)	1258 [21–146] 46.4% Mean age^b^ = 44,9	Veterans (8) Active-duty personnel or civilian and veterans (2) Civilians with PTSD (12)	SKY (2) KrY (2) KY (1) TIHY (1)		7 days—6 months 2/week—daily 7—20 sessions 1 h – 4.4 h	CAPS (3) PCL (5) ies (1) DTS (1) 17 scales for additional measures	WLC (2) OF (1) ACG (2) SWHE (1)	All studies have large within-group effects and 4/6 RCTs significant moderate to large effect size between-group Acceptable and feasible intervention
					Mindfulness (9): MBSR (4), MR (2), MBX (1), telehealth mindfulness (1), PCBMT (1) Relaxation (7)		4 – 16 weeks Weekly – 2/week 3—16 sessions 20 min—7 h	CAPS (10), PCL (10) IES (3) SI-PTSD (1) PTS-T (1) PTSDSS (2) PSS-SR (1) 17 scales for additional measures	WLC (3) TAU (3) PCGT (2) ACG (2) DI (1) EMDR (1) CBT (1) Telehealth psychoeducation (1) Others (4)	Mind–body therapies have encouraging evidence but still have methodologic weaknesses Not enough studies have evaluated the secondary outcomes
**Sciarrino et al. 2017** **USA** [32]	SR	7 (7)	391 [22–100] n.r n.r	Military (2) Veterans and civilian women (1) Civilians with PTSD (4)	SKY (1) KrY (1) KY (1) SatY (1) TSY (1) Hatha yoga (1) MBX + yoga postures (1)		5 days – 16 weeks Monthly – daily 10—32 sessions 1 h – 4.4 h	PCL (4) CAPS (2) PDS (1)	WLC (3) No treatment (2) SWHE (1) Demobilization program (1)	5/7 RCTs found significant results in favor of yoga compared to control

^a^Children’s samples did not concern yoga interventions, ^b^ Incomplete data, ^c^ Includes a three arms study, ^d^ Yoga is considered as meditation here, *CI* Confidence intervals, *ES* Effect-size, *MA* Meta-analysis, *MS* Multiple sclerosis; n.r: not reported, *QOE* Quality of evidence, *QOL* Quality of life, *SR* Systematic review, *SMD* standardised mean difference

PTSD SCALES: *BRUMS* Brunel mood scale rating, *CAPS* Clinician administered PTSD scale, *DES* Dissociative experience scale, *DT*S Davidson trauma scale, *HTQ* Harvard trauma questionnaire, *IES* Impact of events scale, *PCL* PTSD checklist (including military, civilian, and 17), *PDS* Post-traumatic stress diagnostic scale, *PVSDS* PTSD checklist, *PSS-I* PTSD symptom scale-Interview, *PSS-SR* Post- traumatic symptom scale-self report. *PTS-T* Posttraumatic stress-total on the detailed assessment of posttraumatic states, *PTSDSS* PTSD symptom severity scale, part of the posttraumatic stress diagnostic scale, *SI-PTSD* PTSD structured interview, *TLEQ* Trauma life events questionnaire

INTERVENTIONS: *KrY* kripalu yoga, *KY* Kundalini yoga, *MBB* Mind–body bridging, *MBSR* Mindfulness-based stress reduction, *MBX* Mindfulness-based stretching and deep breathing exercise, *MR* Mantram repetition, *PCBMT* Primary care brief mindfulness training, *SatY* Satvananda yoga, *SKY* Sudarshan kriya yoga, *TI(H)Y* trauma-informed (hatha) yoga, *TMS* Transcranial magnetic stimulation, *TSY* Trauma-sensitive yoga

CONTROL GROUPS: *ACG* Assessment control group, *CBT-TF* Cognitive-behavioural therapy with a trauma focus, *CBT* Cognitive behavioral therapy, *DI* Delayed-intervention control group, *EMDR* Eye movement desensitization and reprocessing, *PC(G)T* Present-centered (group) therapy, *SWHE* Supportive women’s health education, *TAU* Treatment as usual, *WLC* Wait-list control

Reported in 7 SRs, age ranged from 18 to 70 [3, 24, 25, 27–29, 31] and sex ratio from 32.9% to 100% of women [24, 25, 27–31] (concerns only the yoga group in the SR of Liu et al. [30]). As regards trauma type, the population was heterogeneous with veterans and active military representing about half of the trauma population in 6 SRs [23–27, 30]. In the other 4 SRs, traumas were various in the civilian sample population (victims of natural disasters, interpersonal violence, patients with treatment-resistant PTSD, inmates’ wives, nurses, patients with multiple sclerosis and PTSD, in-patients in psychiatric unit) [3, 28, 31, 32], and trauma type were not specified in one review [29]. Gallegos et al. [26], Hilton et al. [27], and Björkman and Ekblom [24] (9 RCTs including 4 of yoga) analyzed the influence of veteran versus non-veteran population [24, 26], and trauma type [27] on PTSD symptoms, and found no significant difference.

Trauma diagnostic was specified in 5 reviews [3, 24, 25, 28, 32] mostly thanks to the Diagnostic and Statistical Manual (DSM), PTSD Checklists (PCL) or Clinician-Administered PTSD Scale (CAPS).

Three SRs included only yoga interventions [25, 28, 32], whereas 8 associated yoga with other interventions: mindfulness, meditation, relaxation interventions, body-mind-spirit interventions, or physical activity (anaerobic, aerobic and/or resistance-based exercises), or other interventions [3, 23, 24, 26, 27, 29–31]. Three of the SRs with mixed interventions provided overall results and reported specific forest plot analysis for yoga intervention [3, 24, 29], while 5 SRs reported specific results and figures of yoga interventions [23, 26, 27, 30, 31]. The 11 SRs included 59 primary studies, of which 13 were only yoga interventions.

One SR specifically focused on trauma-sensitive yoga [28], while the others had no restriction on the type of yoga. Eight different types of yoga were assessed: trauma-informed yoga [3, 24–27, 29–32], Kripalu yoga [3, 24–27, 29–32], Kundalini yoga [24–27, 29, 31, 32], Sudarshan Kriya yoga [25, 29, 31, 32], Satyananda yoga [25, 29, 32], Hatha yoga [29, 32], online yoga [24, 29], Mindfulness-based stretching and deep breathing exercise [26, 32], while the type of yoga was not specified in one SR [23].

The duration of the yoga interventions ranged from 5 days to 6 months, with daily to monthly practice including 7 to 32 sessions lasting from 1 to 4.4 h. A sub-group analysis by Zhu et al. [29] showed that a duration of 8 to 16 weeks with 60 to 150 min per session would increase the benefits of mindfulness exercises on PTSD. Four reviews reported that yoga was delivered in group or individual sessions [24, 26, 30, 31].

### Outcomes

#### PTSD symptoms

Fifteen scales were used to assess PTSD symptom changes, of which the most recurrent were CAPS for 10 SRs and PCL for 9 SRs. Seven SRs reported significant effects in favor of yoga with small to moderate effect size compared to control [3, 23–27, 29] and 1 review indicated no significant changes [28]. Bjorkman and Ekblom [24] found no significant difference between low- and high- intensity activities (yoga vs aerobic and/or resistance training). Liu et al. [30] reported that 2 out of 3 RCTs had significant PTSD symptoms relief after the yoga intervention. Sciarrino et al. [32] reported that 5 out of 7 RCTs had significant positive results after yoga compared to control condition. Niles et al. [31] reported that 4 out of 6 RCTs had significant between group-effects in favor of yoga, with moderate to large effect-size.

#### Secondary outcomes, follow-up, safety, adherence, cost

Yoga effectively reduced depression in 4 SRs (3 to 6 RCTs of yoga) [3, 24, 27, 29] while not in one (3 RCTs of yoga) [28]. No significant difference in anxiety was reported in two SRs (7 RCTs including 3 on yoga) [24, 27], while one found positive significant effects in 5 RCTs of mind–body therapies on anxiety (4 RCTs of yoga) [29]. One SR reported significant sleep amelioration with moderate effect size (4 RCTs with 2 of yoga) [24], while another did not [3].

Niles et al. [31] reported that 3 out of the 6 yoga interventions showed that symptom relief was maintained from 1 month to 1 year after completion of yoga intervention. In the SR of Liu et al. [30], 1 yoga RCT [33] reported no significant CAPS score changes after 1 month of follow-up.

Cramer et al. [34] reported no adverse events in two RCTs, while exacerbations of preexisting breathing problems were observed in one RCT. Hilton et al. [27] and Rosenbaum et al. [3] did not report adverse events in yoga interventions.

Two SRs reported that attrition was between 0 and 62% [30, 31].

None of the SRs evaluated the cost of interventions.

### Comparators

Control groups consisted of usual treatment, wait-list, active treatment and/or non-active treatment, delayed intervention control group and/or assessment control. The three SRs performing a meta-analysis showed no significant difference between the active and non-active control group [24, 26, 27].

#### Methodological quality, risk of bias, quality of evidence and funding

Seven SRs reported risk of bias tool assessment using the Cochrane risk of bias tool [25, 26], the Grading of Recommendations, Assessment, Development and Evaluation (GRADE) [23, 27], the Delphi list [30], the modified Physical Therapy Evidence Database scale (PEDro) [29] or the Swedish agency for Health Technology Assessment and Assessment of Social Services (SBU) [24]. As they reported unclear to high risk of bias in their analyses, level of evidence was downgraded to very low [23, 25, 27] or moderate quality [24].

AMSTAR-2 assessment is presented in Table 2. Two authors agreed at 97% (NL and MGG) in their ratings. The mean score was 7/16, with a minimum of 2 [30] and a maximum of 12 [27]. None of the included reviews presented the fundings of included RCTs. Cramer’s review was rated as moderate [25] in overall confidence, 3 as low [26, 27, 29] and 7 reviews as critically low [3, 23, 24, 28, 30–32].

**Table 2:** Methodological quality assessment

**AMSTAR 2 criterion**^**a**^	**USE PICO**	**METHOD**	**INCLUSION**	**SEARCH STRATEGY**	**SELECTION × 2**	**EXTRACTION × 2**	**EXCLUSIONS**	**DESCRIPTION**	**ROB RCT**
Systematic review	1	2	3	4	5	6	7	8	9
Niles, 2018 [31]	Y	P	Y	P	N	N	N	P	N
Rosenbaum 2015 [3]	Y	P	N	P	Y	N	N	P	P
Cramer 2018 [25]	Y	P	N	P	Y	Y	Y	Y	Y
Hilton 2017 [27]	Y	Y	Y	Y	Y	Y	N	Y	Y
Gallegos 2017 [26]	Y	P	Y	P	Y	Y	N	Y	Y
Sciarrino 2017 [32]	Y	N	Y	P	N	N	N	P	N
Kysar- Moon 2021 [28]	Y	N	Y	P	N	N	N	Y	N
Björkman 2021 [24]	Y	P	N	P	Y	N	N	P	P
Liu 2018 [30]	Y	P	N	P	N	N	N	P	P
Bisson 2020^b^ [23]	Y	P	Y	Y	N	Y	N	N	Y
Zhu 2021 [29]	Y	Y	N	P	Y	N	N	Y	P
% of "No"	0	18	45	0	45	63	91	9	27

**AMSTAR 2 criterion**^**a**^	**FUNDING**	**MA METHOD**	**MA ROB IN RESULTS**	**ROB DISCUSSION**	**HETEROGENEITY**	**MA ROB DISCUTED**	**COI & FUNDINGS**		**Overall confidence in the results of the reviews**
Systematic review	10	11	12	13	14	15	16	Rating (/16)	
Niles, 2018 [31]	N	n.a	n.a	N	N	n.a	Y	3	Critically low
Rosenbaum 2015 [3]	N	N	N	N	Y	Y	N	5	Critically low
Cramer 2018 [25]	N	Y	Y	Y	N	Y	Y	11	Moderate
Hilton 2017 [27]	N	Y	Y	N	Y	N	Y	12	Low
Gallegos 2017 [26]	N	N	N	Y	Y	Y	Y	11	Low
Sciarrino 2017 [32]	N	n.a	n.a	N	N	n.a	Y	3	Critically low
Kysar- Moon 2021 [28]	N	Y	N	N	Y	N	Y	6	Critically low
Björkman 2021 [24]	N	Y	Y	N	Y	Y	Y	7	Critically low
Liu 2018 [30]	N	n.a	n.a	Y	N	n.a	N	2	Critically low
Bisson 2020^b^ [23]	N	N	Y	Y	N	N	Y	8	Critically low
Zhu 2021 [29]	N	Y	Y	Y	Y	Y	N	9	Low
% of "No"	100	9	27	54	45	27	27	Mean score = 7	

^a^Items 9 and 11 are presented without 9.1/9.2 and 11.1/11.2 distinction as there is only RCT in this overview

^b^Data available in previous publications [7, 35, 36]

°AMSTAR 2 critical domains, *Y* Yes, *P* Partially yes, *N* No, *n.a* not applicable

Rating overall confidence in the results of the review:

High: No or one non-critical weakness. The systematic review provides an accurate and comprehensive summary of the results of the available studies that address the question of interest

Moderate: more than one non-critical weakness. The systematic review has more than one weakness but no critical flaws. It may provide an accurate summary of the results of the available studies that were included in the review

Low: one critical flaw with or without non-critical weaknesses. The review has a critical flaw and may not provide an accurate and comprehensive summary of the available studies that address the question of interest

Critically low: more than one critical flaw with or without non-critical weaknesses. The review has more than one critical flaw and should not be relied on to provide an accurate and comprehensive summary of the available studies

## Discussion

This overview was aimed at gathering recent evidence considering the potential positive effects of yoga on PTSD symptoms by including 11 SRs with additional meta-analysis in 8 of them. All in all, yoga provides benefits for PTSD symptoms, although the quality of evidence is low, given the heterogeneity and methodological concerns.

In 13 SRs dated from 2005 to 2013, the overview by Macy et al. [16] reported that yoga intervention could relieve negative outcomes of trauma (depression, anxiety, and PTSD). However, as only 30% of the primary studies were RCTs, their main recommendation was to increase the level of evidence by applying randomized control trial design. Close to ten years later, our overview included only SRs with RCTs, and additionally assessed quality of evidence using the AMSTAR-2 tool. The current overview highlights the fact that 3 SRs without meta-analysis [30–32] and 7 SRs with meta-analysis [3, 23–27, 29] reported significant positive effects of yoga compared to control interventions in PTSD symptoms with small to moderate size effect, while one failed to report significant benefit of yoga [28]. The quality of evidence was rated as very low to moderate in 4 SRs [23–25, 27], and 6 SRs concluded that yoga could only be considered as an adjunctive treatment to conventional approach [3, 24–26, 29, 30]. For the sake of the PTSD patient, our overview supported the use of yoga as a complementary approach in clinical practice, while we recommend performance of RCTs comparing yoga with and without conventional treatment, the objective being to delineate the effectiveness of yoga intervention more precisely in the PTSD population.

As aforementioned, PTSD symptoms can originate from several traumas. Referring to PTSD patients as a homogeneous population could be considered as a restrictive point of view. Even though our results, as previously reported [16], failed to demonstrate that different PTSD origins could cause more or fewer benefits, it bears mentioning that psychological and social factors might influence response to therapy, as previously demonstrated in the low back pain population [37–39]. For instance, the process of PTSD management in male veterans and active military persons, predominantly represented in our overview [24, 26, 27], cannot be equated to sexual abuse of women. Far from placing populations in opposition, specific yoga therapy programs might strengthen the effectiveness of this approach. Social factors, such as social gradient of health [38], professional status [39] or gender, should be taken into account and systematically assessed to guide yoga teachers to modulate their programs. Considering the numerous types of yoga, including the 8 identified in our overview, it is safe to suggest that a multidisciplinary approach, including Acceptance and Commitment Therapy (ACT) [40] with medical and yoga teachers would help to shed light on this specific issue. So as to standardize therapeutic approach we recommend determining a specific yoga program dedicated to people presenting with PTSD associated with specific modules for the different types of PTSD. In addition, the duration and frequency of yoga therapy sessions should be considered. Yoga therapy could be modeled on other complementary approaches such as mindfulness [41, 42] (e.g., Mindfulness Based Stress Reduction or Mindfulness-Based Cognitive therapy) and clinical hypnosis [43], which recommend practicing for at least 8 weeks with a professional in order to achieve significant effects on anxiety or pain, potentiated by home self-practice [44, 45]. Subgroup analysis by Zhu et al. [29] showed that 8 to 16 weeks with 60 to 150 min per session improved the benefits of mindfulness exercises on PTSD symptoms. Future research is needed to determine the “dose–response” relationship for yoga therapy delivered to PTSD patients.

Based on three main pillars (physical postures, breathing techniques and meditation), yoga therapy is promising insofar as it helps to manage PTSD symptoms. In addition to subjective benefits, clinical research has investigated neuroimaging in different populations to support yoga-related neuroplasticity. In a recent review, van Aalst et al. [46] synthesized neurobiology advances determined from a neuroimaging framework. First, the authors stated that despite heterogeneous practice of yoga and target assessment, cerebral structural and functional changes were consistent in the 34 included studies. Morphological studies showed an increase in regional grey matter density or volume in yoga practitioners compared to controls, with a higher grey matter localized at the insula [47–49] identified as central in interoceptive body awareness and empathy [50, 51]. In addition, studies reported grey matter volume increase in hippocampus [47, 48, 52, 53] and decrease in the amygdala, which was likely associated with lower experienced stress due to yoga practice [54–57]. Furthermore, yoga meditation showed increased frontal region activation of several frontal regions [58–61], known to be key to decision-making [62, 63], motor control [64, 65] and sustained attention [66]. In line with this result, one study reported that yoga meditation significantly increased dopaminergic release in the ventral striatum [67], playing an important role in the circuitry underlying goal-directed behaviors, changes in affective states [68], and in the reward/motivation circuitry [69]. All in all, both bottom-up (physical posture and breathing) and top-down (focused attention) mechanisms of action were involved in yoga therapy [70]. While these findings have not been collected in PTSD patients, the consistency of the results suggest that similar morphological and functional pathways are involved after yoga therapy in PTSD patients. Specific neuroplasticity after yoga therapy in PTSD patients has to be determined.

### Limitations

Although providing new insight, this overview has several limitations. First, the high overlap rate (CCA of 21%) could have over-estimated conclusions from SRs including similar studies. In addition, the sample size of included studies was unequally distributed across the SRs (from 3 to 22) and the overall low methodological quality of included SRs (7 SR reported high risk of bias of included studies [23–27, 29, 30]) should nuance our conclusion. Our overview included SRs focusing on PTSD symptoms but avoiding considering secondary outcomes such as pain and physical activity [71] that have been shown to be associated with psychological distress [72–75]. The heterogeneity of the yoga practices did not allow us to highlight which type of yoga would potentially the best approach for the PTSD population. Finally, future studies should include long- term follow-up periods to better establish yoga therapy efficacy.

## Conclusion

The current overview, including 11 SR with RCT studies, highlighted promising results of yoga effectiveness for management of PTSD symptoms. In collaboration with clinicians and psychotherapists, this overview suggests a need for specific yoga programs taking into account social factors and having a standardized duration of 8 weeks (or more), the objective being to assess not only PTSD symptoms, but also secondary outcomes such as pain and physical activity. Future studies should include long-term follow-up duration and neuroimaging to specifically delineate efficacy and neuroplasticity in PTSD patients.

## Supplementary Information

**Additional file 1. **Search strategies for databases used in the review.

**Additional file 2. **Characteristics of excluded studies.

## Data Availability

The datasets used and/or analysed during the current study available from the corresponding author on reasonable request.

## Abbreviations

AMSTAR-2: A measurement tool to assess systematic reviews
CAPS: Clinician-administered PTSD scale
CCA: Corrected covered area
DSM: Diagnostic and statistical manual
GRADE: Grading of recommendations, assessment, development and evaluation
PCL: PTSD checklists
PRIO-harms: Preferred Reporting Items for Overviews of SR Including Harms
PTSD: Post-traumatic stress disorder
RCT: Randomized controlled trial
SBU: Swedish agency for health technology assessment and assessment of social services
SR: Systematic review
