# Supplementary material for: Management of post-traumatic stress disorder symptoms by yoga: an overview
Source: BMC Complement Med Ther. 2023 Jul 21;23:258. doi: 10.1186/s12906-023-04074-w (PMC10360332; doi:10.1186/s12906-023-04074-w)
Supplement: Supplementary file 1 — Additional file 1. Search strategies for databases used in the review. [file 12906_2023_4074_MOESM1_ESM.docx]

**Appendix 1: search strategies for databases used in the review**

1. **Medline (Pubmed):**

[Search date March 10^th^ 2022]

The search string was: (((PTSD [Title/Abstract]) OR (posttraumatic stress disorder [Title/Abstract]) OR (post-traumatic stress disorder [Title/Abstract]) OR (posttraumatic neuroses [Title/Abstract]) OR (post- traumatic neuroses [Title/Abstract])) AND (Yoga [Title/Abstract])) AND (systematicreview[Filter])

**19 potential articles were retrieved**

1. **PEDro:**

[Search date March 10^th^ 2022]

The search string was:

- Substract & title: yoga AND PTSD
- Therapy:
- Problem:
- Body part:
- Subdiscipline:
- Topic:
- Method: systematic review
- Match all search terms (AND)

**8 potential articles were retrieved**

1. **Cochrane Library:**

[Search date March 10^th^ 2022]

## The search string was: yoga in Title Abstract Keyword AND posttraumatic stress disorder in All Text - (Word variations have been searched)

**2 potential articles were retrieved**

1. **Scopus:**

[Search date March 10^th^ 2022]

The search string was: TITLE-ABS-KEY (yoga) AND TITLE-ABS-KEY (ptsd OR posttraumatic AND stress AND disorder OR post AND traumatic AND stress AND disorder) AND (EXCLUDE (DOCTYPE, “no”) OR EXCLUDE (DOCTYPE, “cp”) OR EXCLUDE (DOCTYPE, “le”))

**131 potential articles were retrieved**

1. **Embase:**

[Search date March 10^th^ 2022]

('posttraumatic stress disorder'/exp OR 'ptsd' OR 'ptsd (posttraumatic stress disorder)' OR 'post-traumatic stress' OR 'post-traumatic stress disorder' OR 'post-traumatic stress disorders' OR 'posttraumatic neurosis' OR 'posttraumatic psychic syndrome' OR 'posttraumatic psychosis' OR 'posttraumatic stress' OR 'posttraumatic stress disorder' OR 'posttraumatic syndrome' OR 'psychosis, posttraumatic' OR 'stress disorders, post-traumatic' OR 'stress disorders, traumatic' OR 'stress, posttraumatic' OR 'trauma and stressor related disorders' OR 'traumatic stress' OR 'traumatic stress disorder' OR 'traumatic stress disorders') AND ('yoga'/exp OR 'yoga' OR 'yogic meditation') AND ('systematic review'/exp OR 'review, systematic' OR 'systematic review')

**40 potential articles were retrieved**

1. **CINHAL:**

[Search date March 10^th^ 2022]

The search string was:
TX yoga AND TX (PTSD or post-traumatic stress disorder OR post-traumatic stress disorder OR posttraumatic neuroses OR post-traumatic neuroses ) AND systematic review

Expansion operators: apply related words and apply equivalent subjects

**71 potential articles were retrieved**
