# Supplementary material for: Management of post-traumatic stress disorder symptoms by yoga: an overview
Source: BMC Complement Med Ther. 2023 Jul 21;23:258. doi: 10.1186/s12906-023-04074-w (PMC10360332; doi:10.1186/s12906-023-04074-w)
Supplement: Supplementary file 2 — Additional file 2. Characteristics of excluded studies. [file 12906_2023_4074_MOESM2_ESM.docx]

**Appendix 2: characteristics of excluded studies**

| **Author** | **Year** | **Title** | **Reason for exclusion** |
| --- | --- | --- | --- |
| **Brinsley et al.** | 2021 | Effects of yoga on depressive symptoms in people with mental disorders: a systematic review and meta-analysis | P: Not only PTSD |
| **Cabral et al.** | 2011 | Effectiveness of yoga therapy as a complementary treatment for major psychiatric disorders: a meta-analysis [with consumer summary] | Only one RCT on PTSD + other pathologies |
| **Cushing et al.** | 2018 | Mind-body therapy for military veterans with post-traumatic stress disorder: A systematic review | SD: not only RCTs |
| **Da Silva et al.** | 2009 | Yoga in the treatment of mood and anxiety disorders: A review | SD: not only RCTs |
| **Kim et al.** | 2013 | Mind-body practices for posttraumatic stress disorder | SD: not only RCTs |
| **Krisanaprakornkit et al.** | 2006 | Meditation therapy for anxiety disorders | Only one RCT on yoga, SR of 2 RCTs |
| **Lee et al.** | 2021 | A Review of Trauma Specific Treatments (TSTs) for Post-Traumatic Stress Disorder (PTSD) | SD: not only RCTs |
| **Longacre et al.** | 2012 | Complementary and alternative medicine in the treatment of refugees and survivors of torture: a review and proposal for action. | I: no article on yoga  SD: not only RCTs |
| **Metcalf et al.** | 2016 | Efficacy of fifteen emerging interventions for the treatment of posttraumatic stress disorder: a systematic review | SD: not only RCTs |
| **Nguyen-Feng et al.** | 2019 | Yoga as an intervention for psychological symptoms following trauma: A systematic review and quantitative synthesis | SD: not only RCTs |
| **Oppizzi et al.** | 2018 | The Effect of Physical Activity on PTSD | SD: not systematic review |
| **Pradhan et al.** | 2016 | Nonconventional interventions for chronic post-traumatic stress disorder: Ketamine, repetitive trans-cranial magnetic stimulation (rTMS), and alternative approaches | SD: not systematic review |
| **Wahbeh et al.** | 2014 | Complementary and Alternative Medicine for Posttraumatic Stress Disorder Symptoms: A Systematic Review. | SD: not only RCTs |
| **Walker et al.** | 2021 | Depression, anxiety, and other mental disorders in patients with cancer in low- And lower-middle-income countries: A systematic review and meta-analysis | SD: not only RCTs |
| **Whitworth et al.** | 2016 | Exercise and Post-Traumatic Stress Disorder in Military Veterans: A Systematic Review. | I: no article on yoga  SD: not only RCTs |
| **Wynn** | 2015 | Complementary and Alternative Medicine Approaches in the Treatment of PTSD | SD: not systematic review |

I: Intervention, O: Outcome, P: Population, RCT: Randomized Controlled Trial, SD: Study Design
